# Physiological and Molecular Traits Associated with Nitrogen Uptake under Limited Nitrogen in Soft Red Winter Wheat

**DOI:** 10.3390/plants10010165

**Published:** 2021-01-17

**Authors:** Suman Lamichhane, Chiaki Murata, Carl A. Griffey, Wade E. Thomason, Takeshi Fukao

**Affiliations:** 1 School of Plant and Environmental Sciences, Virginia Tech, Blacksburg, VA 24061, USA; sumanl7@vt.edu (S.L.); cgriffey@vt.edu (C.A.G.); wthomaso@vt.edu (W.E.T.); 2 Texas A&M Agrilife Research, Beaumont, TX 77713, USA; 3 Department of Bioscience and Biotechnology, Fukui Prefectural University, Eiheiji, Fukui 910-1195, Japan; s1721043@g.fpu.ac.jp

**Keywords:** *Triticum aestivum*, nitrogen uptake, nitrogen transporters, nitrogen use efficiency

## Abstract

A sufficient nitrogen (N) supply is pivotal for high grain yield and desired grain protein content in wheat (*Triticum aestivum* L.). Elucidation of physiological and molecular mechanisms underlying nitrogen use efficiency (NUE) will enhance our ability to develop new N-saving varieties in wheat. In this study, we analyzed two soft red winter wheat genotypes, VA08MAS-369 and VA07W-415, with contrasting NUE under limited N. Our previous study demonstrated that higher NUE in VA08MAS-369 resulted from accelerated senescence and N remobilization in flag leaves at low N. The present study revealed that VA08MAS-369 also exhibited higher nitrogen uptake efficiency (NUpE) than VA07W-415 under limited N. VA08MAS-369 consistently maintained root growth parameters such as maximum root depth, total root diameter, total root surface area, and total root volume under N limitation, relative to VA07W-415. Our time-course N content analysis indicated that VA08MAS-369 absorbed N more abundantly than VA07W-415 after the anthesis stage at low N. More efficient N uptake in VA08MAS-369 was associated with the increased expression of genes encoding a two-component high-affinity nitrate transport system, including four *NRT2*s and three *NAR2*s, in roots at low N. Altogether, these results demonstrate that VA08MAS-369 can absorb N efficiently even under limited N due to maintained root development and increased function of N uptake. The ability of VA08MAS-369 in N remobilization and uptake suggests that this genotype could be a valuable genetic material for the improvement of NUE in soft red winter wheat.

## 1. Introduction

Wheat (*Triticum aestivum* L.) is a major staple crop that provides nutrition to more than one-third of the world’s population. In 2018, wheat was cultivated on approximately 214 million hectares of land across the world [1], making the crop more widely grown than any other cereal crops. The application of nitrogen (N) fertilizers is essential for high grain yield and desired grain protein content in wheat. Out of the total N applied globally, 18% is used for wheat, making it the largest amount of N applied to any crop [2]. However, only 30–50% of N supplied is taken up by the plants [3,4]. The non-absorbed N is lost to water bodies and atmosphere through leaching, surface runoff, and denitrification, resulting in soil, water, and air pollution [5]. Improvement of wheat’s ability to absorb N should mitigate these environmental issues and reduce the production costs for this economically important crop. 

Plants absorb N from the soil through their roots, mainly in the form of nitrate and ammonium. A set of plasma-membrane localized transporters is responsible for the uptake of nitrate and ammonium into the root cells. Nitrate uptake is mediated by two major families of transporters: NITRATE TRANSPORTER 2 family (NRT2) and NITRATE TRANSPORTER 1/PEPTIDE TRANSPORTER family (NPF) (formerly called NRT1) [6]. NRT2 transporters generally have a high affinity for nitrate and are induced under nitrate-limiting conditions [7,8]. An exception is rice (*Oryza sativa*) NRT2.4, which displays a dual affinity [9]. In Arabidopsis (*Arabidopsis thaliana*), seven *NRT2*s have been identified [10]. Of these genes, *AtNRT2.1*, *AtNRT2.2*, *AtNRT2.4*, and *AtNRT2.5* are responsible for the uptake of approximately 95% of total nitrate under limited N conditions [11,12]. In rice, four *NRT2* genes have been found [13,14,15]. Interestingly, all NRT2s except AtNRT2.1 and OsNRT2.3b (a splice form of OsNRT2.3) require NITRATE ASSIMILATION RELATED PROTEIN 2 (NAR2) to transport nitrate [15], and therefore this process is designated as a two-component nitrate transport system. 

Plants also transport nitrate using NPFs, which generally have low nitrate affinities [16]. Exceptions are AtNPF6.3 in Arabidopsis and OsNPF6.5 in rice, which exhibit dual affinities [17,18]. Besides nitrate, NPFs transport divergent substrates including peptides, hormones, and glucosinolate [19]. Unlike *NRT2*s, most *NPF* members are not responsive to low N [7]. Although a huge number of *NPF*s (Arabidopsis, 53 genes; rice, 93 genes) have been identified, only limited members have been functionally characterized to date [6]. 

Another major N source is ammonium, which is absorbed by ammonium transporters (AMTs) in plants. Six *AMT*s have been identified in Arabidopsis, three of which (*AtAMT1;1*, *AtAMT1;2*, and *AtAMT1;3*) are involved in the uptake of approximately 90% of ammonium [20]. In rice, 10 *AMT* genes have been found, of which *OsAMT1.1*, *OsAMT1.2*, and *OsAMT1.3* play a significant role in ammonium absorption in roots [21]. Altogether, the functional importance of N transporters has been characterized mostly in Arabidopsis and rice, but their roles in N uptake under limited N supply are still elusive in other plants, including wheat. 

Our previous study involved a comparative analysis of two soft red winter wheat genotypes, VA08MAS-369 and VA07W-415, with contrasting nitrogen use efficiency (NUE) [22]. High NUE in VA08MAS-369 was associated with accelerated senescence and N remobilization in flag leaves after the anthesis stage under N limitation. Consistently, genes and enzymes involved in N remobilization were highly activated in VA08MAS-369 relative to VA07W-415 at low N. In this study, we compared these previously characterized wheat genotypes regarding the capability of N uptake, characterizing physiological and molecular traits responsible for efficient N absorption under limited N.

## 2. Results

### 2.1. Effect of N Supply on Grain Yield and NUE Indexes

**Figure 1:**
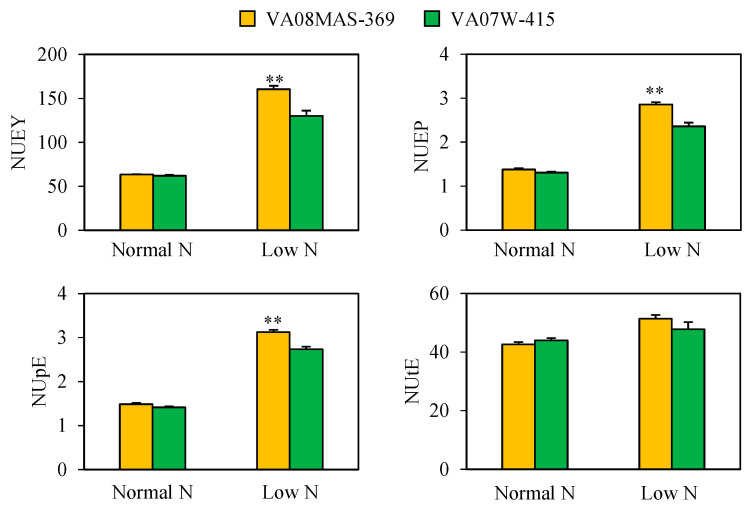
Nitrogen Use Efficiency (NUE) indexes of VA08MAS-369 and VA07W-415 grown in a greenhouse under normal and low N. NUEY, Nitrogen Use Efficiency for Yield = Grain yield/Total N supplied. NUEP, Nitrogen Use Efficiency for Protein = Grain protein content/Total N supplied. NUpE, N Uptake Efficiency = Aboveground N content/Total N supplied. NUtE, Nitrogen Utilization Efficiency = Grain yield/Aboveground N content. Data represent means ± SE (n = 4). ** *p* < 0.01.

This study compared the previously characterized soft red winter wheat genotypes, VA08MAS-369 (high NUE) and VA07W-415 (low NUE), under normal and low N to determine whether they exhibit distinct N uptake efficiency. These genotypes were grown in a greenhouse under identical growth conditions and N treatments to those used in our previous study [22]. Grain yield and yield parameters in the two genotypes were consistent in the previous and present greenhouse experiments. For example, grain yield, spike number per plant, and grain number per plant were larger in VA08MAS-369 than VA07W-415 at low N (Figure S1). An exception was grain number per spike. This parameter was larger in VA08MAS-369 in the present study, but the opposite was true in the previous study [22]. We also verified that NUE for yield (NUEY) and NUE for protein (NUEP) were greater in VA08MAS-369 than VA07W-415 under N limitation (Figure 1), which agreed with the previous study. These results confirmed the reproducibility of the two independent experiments. 

NUEY can be separated into nitrogen uptake efficiency (NUpE) and nitrogen utilization efficiency (NUtE), with NUEY being the product of the two [23]. NUpE is the efficiency of a plant’s absorption of the available N from the soil and is defined as the aboveground N content divided by the total N supplied. NUtE describes how efficiently absorbed N is utilized for grain production and is determined by grain yield divided by aboveground N content. In this study, no significant differences in NUpE and NUtE were detected between the two genotypes under normal N (Figure 1). However, at low N, NUpE was significantly larger in VA08MAS-369 than VA07W-415, indicating that high NUEY in VA08MAS-369 is attributable to its ability to uptake N efficiently, even under N limitation. 

### 2.2. Effect of N Input on Root Morphological Parameters 

**Figure 2:**
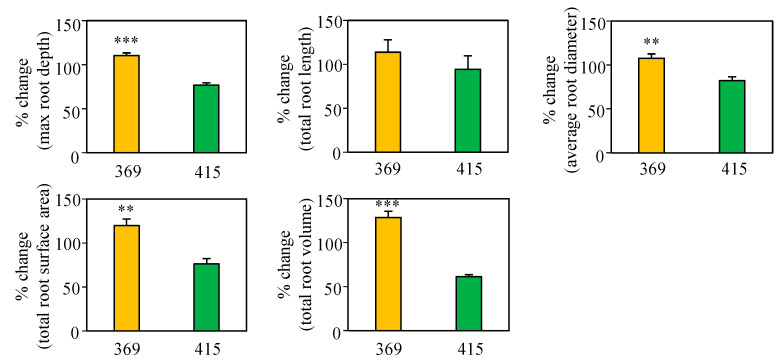
Root morphology of VA08MAS-369 and VA07W-415 grown under normal and low N. Four-day-old seedlings were pre-cultured using the cigar roll method for 3 days under normal N. Then, seedlings were incubated in the same system under normal or low N for additional 6 days. Root morphology parameters were quantified using WinRhizo 2017a. Data represent means ± SE (*n* = 12). 369; VA08MAS-369. 415; VA07W-415. ** *p* < 0.01, *** *p* <0.001.

Root morphology is a critical trait that influences efficient N absorption under low N. To compare root development in the two genotypes under regular and low N, we investigated root morphology parameters using the well-established cigar roll method [24] (Figure 2). Our analysis revealed that maximum root depth, average root diameter, total root surface area, and total root volume were reduced by low N supply in VA07W-415, but these parameters were maintained or even increased in VA08MAS-369. These results demonstrate that VA08MAS-369 can maintain root growth under N deficiency, contributing to high NUpE in this genotype.

### 2.3. Time-Course Observation of N Contents under Normal and Low N

**Figure 3:**
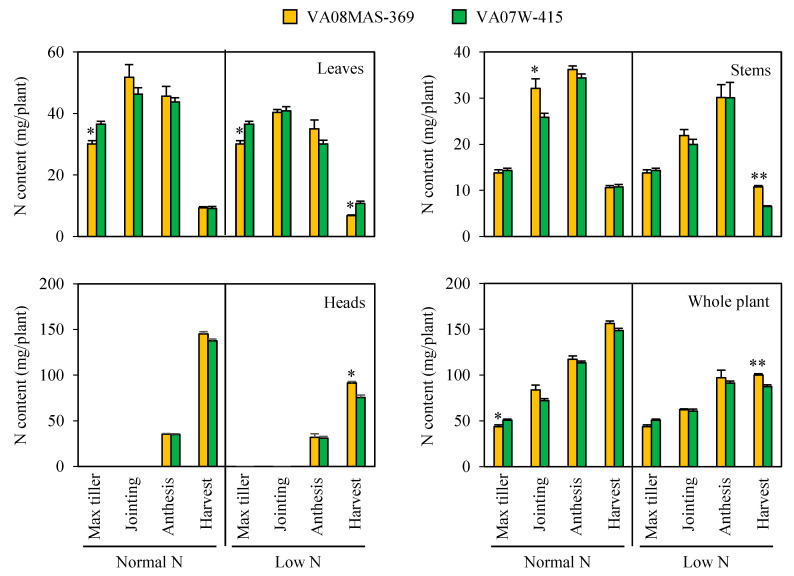
Time-course observation of N contents in leaves, stems, and heads of plants grown in a greenhouse under normal and low N. Leaves, stems, and heads (if available) were harvested at the specified developmental stages and exposed to N content analysis. Data represent means ± SE (*n* = 4). * *p* < 0.05, ** *p* < 0.01.

To determine the developmental stages when efficient N uptake occurs, we monitored N contents in leaves, stems, heads, and a whole plant at different growth stages, under normal and low N (Figure 3). At regular N input, the N contents in leaves, stems, and a whole plant were mostly similar in the two genotypes, especially at latter developmental stages. Under low N, the N contents in stems, heads, and a whole plant were significantly higher in VA08MAS-369 at harvest. In contrast, the leaf N content was lower in VA08MAS-369 than VA07W-415, presumably due to more efficient N remobilization from leaves to grains in the high NUE accession, VA08MAS-369. Overall, these results suggest that higher NUpE in VA08MAS-369 at low N may be caused by larger N uptake at the post-anthesis stage in this genotype. 

### 2.4. Effect of N Input on Grain Yield, NUE Indexes, and N Contents in Plants Grown in a Hydroponic Culture System 

**Figure 4:**
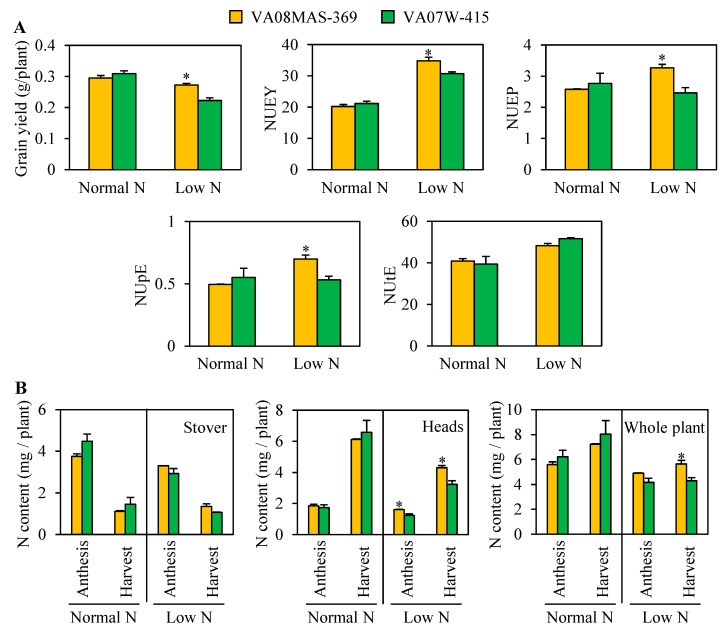
Grain yield, NUE indexes (**A**), and N contents (**B**) in plants grown in a hydroponic system under normal and low N. Data represent means ± SE (*n* = 3). * *p* < 0.05.

To determine the mRNA accumulation of genes associated with N uptake in roots, intact root tissues are required. For this purpose, we grew wheat plants using a hydroponic culture system under regular and low N conditions. This study confirmed that grain yield, NUEY, NUEP, and NUpE were larger in VA08MAS-369 than VA07W-415 under N limitation, consistent with the results obtained in the greenhouse (Figure 1, Figure 4A and Figure S1). No significant differences in the stover N content were detected between the two genotypes at the anthesis and harvest stages, regardless of N conditions (Figure 4B). The head N content was higher in VA08MAS-369 than VA07W-415 at anthesis and harvest under N limitation. The whole plant’s N content was greater in VA08MAS-369 at harvest under low N. These results confirmed that N accumulation patterns, as well as grain yield and NUE indexes, were consistent under greenhouse and hydroponic conditions. 

### 2.5. Transcript Accumulation of Nitrate and Ammonium Transporters under Regular and Low N

**Figure 5:**
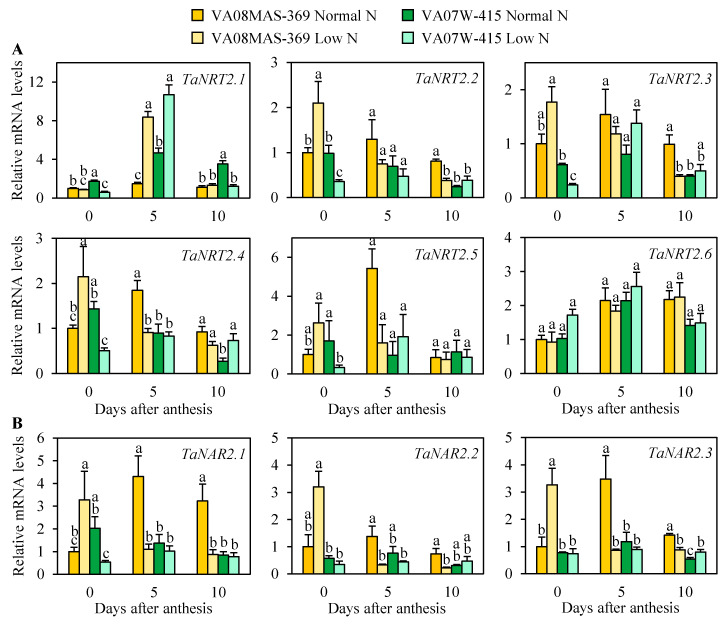
Relative mRNA levels of all *NRT2* (**A**) and *NAR2* (**B**) genes, members of a two-component high-affinity nitrate uptake system, in roots of plants grown in a hydroponic system under normal and low N. Root samples were harvested at the specified time points and subjected to qRT-PCR analysis. Data represent means ± SE (*n* = 3). Bars not sharing the same letter are statistically significant (*p* < 0.05).

The wheat genome encodes six *NRT2* and three *NAR2* genes [25], members of a two-component high-affinity nitrate uptake system. Using root tissues collected from plants grown in a hydroponic system, the mRNA accumulation of these nine genes was monitored by qRT-PCR. Of the six *NRT2* genes in wheat, *TaNRT2.1*, *TaNRT2.2*, and *TaNRT2.4* were significantly induced in response to low N in roots of VA08MAS-369 and/or VA07W-415 at the post-anthesis stage (Figure 5A). Under N limitation, the levels of *TaNRT2.2*, *TaNRT2.3*, *TaNRT2.4*, and *TaNRT2.5* mRNAs were significantly higher in VA08MAS-369 than VA07W-415 at the anthesis stage. In Arabidopsis and rice, most NRT2s require NAR2 to be functional as nitrate transporters [15]. This study indicates that two of the wheat *NAR2* genes, *TaNAR2.1* and *TaNAR2.3*, were upregulated by low N in VA08MAS-369, and all the three *NAR2* mRNAs were more abundant in the high NUE genotype under N limitation at the anthesis stage (Figure 5B).

**Figure 6:**
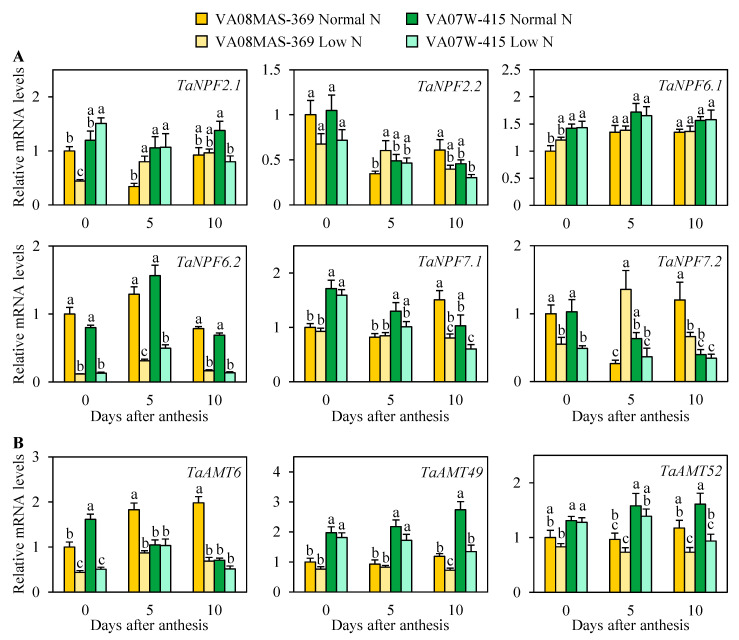
Relative mRNA levels of representative low-affinity nitrate transporters *NPF*/*NRT1*s (**A**) and ammonium transporters *AMT*s (**B**) in roots of plants grown in a hydroponic system under normal and low N. Root samples were harvested at the specified time points and subjected to qRT-PCR analysis. Data represent means ± SE (*n* = 3). Bars not sharing the same letter are statistically significant (*p* < 0.05).

Unlike the high-affinity NRT2-NAR2 nitrate uptake system, the low-affinity NPF system consists of a large gene family in plants (e.g., Arabidopsis, 53 genes; rice, 93 genes) [6]. In wheat, 113 *NPF* genes (one from each homeologous group) have recently been identified [26]. This study investigated the mRNA levels of representative *TaNPF* genes (Figure 6A). Of the six *TaNPF* genes surveyed, *TaNPF2.1*, *TaNPF2.2*, and *TaNPF7.2* were upregulated by low N in VA08MAS-369 5 days after anthesis. *TaNPF6.2* was considerably downregulated in response to N limitation in both genotypes at all time points. *TaNPF7.2* mRNA was more abundant in VA08MAS-369 than VA07W-415 under N deficient conditions 5 and 10 days after anthesis, consistent with the N accumulation, NUE, and grain yield data (Figure 1, Figure 3, Figure 4, and Figure S1). By contrast, the transcript levels of *TaNPF2.1*, *TaNPF6.2*, and *TaNPF7.1* were lower in the high NUE genotype relative to the low NUE genotype at the anthesis stage or 5 days after anthesis under limited N, inconsistent with the downstream phenotypes.

Another major N form, ammonium, is absorbed by ammonium transporters (AMTs). The Arabidopsis and rice genomes contain 6 and 10 *AMT* genes, respectively [20,21]. It is still unclear how many *AMT* genes are present in the wheat genome. This study analyzed the expression of representative *AMT* genes (Figure 6B). None of the *AMT* genes surveyed were induced by N limitation. However, the levels of *TaAMT49* and *TaAMT52* transcripts were lower in VA08MAS-369 than VA07W-415 at low N. 

## 3. Discussion

This study compared the two previously characterized soft red winter wheat genotypes regarding N absorption capability under normal and limited N. Our growth experiment revealed that high NUEY in VA08MAS-369 at low N resulted from high NUpE in this genotype (Figure 1). Although NUpE is not a component of NUEP, it can be anticipated that more efficient N uptake (i.e., high NUpE) can contribute to more efficient N accumulation in grains (i.e., NUEP) under N limitation.

This study also determined the developmental stage at which VA08MAS-369 absorbs N more abundantly than VA07W-415 (Figure 3). Under N limitation, VA08MAS-369 displayed greater N accumulation in stems, heads, and a whole plant at harvest, although no genotypic differences in N accumulation were observed in these tissues at earlier stages. This result indicates that efficient N uptake at the post-anthesis stage is critical for high NUpE in VA08MAS-369. Under hydroponic conditions, greater N accumulation was observed slightly earlier (i.e., the anthesis stage) in heads, but the whole plant N accumulation data were consistent in our greenhouse and hydroponic experiments (Figure 3 and Figure 4B). In contrast to the stem, head, and whole plant data, the leaf N content was lower in VA08MAS-369 than VA07W-415 at low N supply (Figure 3). This may result from more active N remobilization from leaves to grains in the high NUE genotype, VA08MAS-369. Indeed, N remobilization in flag leaves was higher in VA08MAS-369 at the post-anthesis stage under limited N [22].

Well-developed plant root systems are pivotal in the efficient acquisition of N from the soil. For example, the ability of N acquisition in maize has been shown to correlate with root length density [27]. Moreover, maize genotypes with fewer crown roots and a deeper root system exhibited significantly higher N uptake from N-limited soils [28]. In rice, high NUE was correlated with greater total root length and root surface area [29]. In this study, we investigated the effect of limited N on root development in the two wheat genotypes with contrasting NUE. Low N suppressed major parameters involved in root development, such as maximum root depth, average root diameter, total root surface area, and total root volume in VA07W-415, but these parameters were maintained or greater in VA08MAS-369 under N limitation (Figure 2). The capability of VA08MAS-369 to continue the development of root systems even at low N must benefit efficient N acquisition. 

Besides root development, the activities of nitrate and ammonium transporters are major factors influencing the acquisition of N from the source. The functional importance of high-affinity nitrate transporters, NRT2s, in nitrate uptake has been revealed by mutant and transgenic analyses in Arabidopsis and rice [14,30,31,32]. Due to their high transport activity at a low nitrate concentration, NRT2s may play a significant role in nitrate absorption under nitrate-deficient conditions. Indeed, AtNRT2.4 and AtNRT2.5 function as major nitrate transporters only when N is limited in Arabidopsis [12,33]. In rice, overexpression of *OsNRT2.3b* and conditional expression of *OsNRT2.1* by the nitrate-inducible *OsNAR2.1* promoter enhanced N uptake, NUE, and grain yield at low N supply [14,32]. The present study revealed that *TaNRT2.2*, *TaNRT2.3*, *TaNRT2.4*, and *TaNRT2.5* (four of the six wheat *NRT2*s) were highly expressed in VA08MAS-369 relative to VA07W-415 under N limitation at the anthesis stage (Figure 5A), consistent with N accumulation patterns in heads and a whole plant (Figure 4B). According to the phylogenetic analysis [25], *TaNRT2.2* and *TaNRT2.3* in wheat are most closely related to *OsNRT2.1* in rice, whereas *TaNRT2.4* and *TaNRT2.5* are most closely related to *OsNRT2.3*. Overall, these results indicate that the wheat *NRT2* genes highly expressed in the high NUE genotype at low N are orthologous to the rice *NRT2* genes that are critical for efficient N uptake and high yield under N-deficient conditions. 

In Arabidopsis and rice, all NRT2s, except for AtNRT2.4 and OsNRT2.3b, require NAR2 for nitrate transport activity [15,33,34]. It is still unclear which NRT2 proteins necessitate NAR2 in wheat, but TaNRT2.4 and TaNRT2.5 might not require NAR2 based on the close phylogenetic relationship between *TaNRT2.4* and *TaNRT2.5* vs. *OsNRT2.3*. In any case, all the wheat *NAR2* genes were highly expressed in VA08MAS-369 under limited N at the anthesis stage (Figure 5B), along with *TaNRT2.2*, *TaNRT2.3*, *TaNRT2.4*, and *TaNRT2.5*. Synchronized mRNA accumulation of *TaNRT2*s and *TaNAR2*s was correlated with the N accumulation data from grains and a whole plant (Figure 4B). In rice, increased expression of *OsNAR2.1*, driven by the *OsNAR2.1* promoter, enhanced the mRNA accumulation of *OsNRT2.1*, *OsNRT2.2*, and *OsNRT2.3a* in roots when nitrate was supplied as an N source [35]. This mechanism may contribute to the synchronized expression of *TaNRT2*s and *TaNAR2*s in wheat. 

Unlike substrate-specific NRT2s, low-affinity nitrate transporters (NPFs) carry divergent substrates including nitrate, peptides, hormones, and glucosinolate [19]. Some NPFs, such as AtNPF8.1, AtNPF8.2, and AtNPF8.3, do not transport nitrate [6]. Nevertheless, several NPFs play a key role in nitrate uptake and transport [36]. Plant *NPF*s are classified into eight clades using phylogenetic analyses. Based on the characterized function of Arabidopsis and rice *NPF* clades/genes [6], we selected representative genes expected to be involved in nitrate transport (Figure 6A). Gene expression analysis of these *NPF*s revealed that only *TaNPF7.2* mRNA was highly induced in VA08MAS-369 at low N, associated with the N accumulation data (Figure 4B). It is unlikely that other *NFP* genes surveyed are responsible for efficient N uptake in the high NUE line because the expression patterns of these *NPF* genes were not correlated with N accumulation patterns. Likewise, we also analyzed the expression of representative *AMT*s (Figure 6B). None of these expression patterns were consistent with the N accumulation patterns. Although the present study analyzed only part of large *NPF* and *AMT* families, the data presented here suggest that the NRT2-NAR2 system could be a larger contributor to N transport under N-deficient conditions as compared with NPFs and AMTs. 

In summary, our physiological and molecular analyses demonstrated that the higher capability of VA08MAS-369 for N absorption is attributable to greater performance in terms of root system development and higher expression of genes associated with the two-component high-affinity nitrate uptake system, especially *TaNRT2.2*-*2.5* and *TaNAR2.1*-*2.3*. The level of N acquisition directly affects wheat yield because proteins (major N compounds) account for approximately 10–15% of the grains. The present study showed that N absorption at and after the anthesis stage is critical for higher N accumulation in grains and grain yield in VA08MAS-369 under limited N. Our previous study demonstrated that enhanced N remobilization in flag leaves benefited maintained grain production at low N supply in VA08MAS-369 [22]. High N uptake in VA08MAS-369 may lead to an increase in vacuolar nitrate storage in flag leaves, eventually contributing to the elevated remobilization of N to heads during grain filling. In addition to nitrate transporter activities, N acquisition is influenced by the capacity to generate a proton gradient because most NRT2s, NPFs, and AMTs are NO_3_^−^/H^+^ or NH_4_^+^/H^+^ symporters [37,38]. It is believed that membrane ATPases and PPases play a significant role in creating a proton gradient during N uptake and storage. Future analysis is required to determine whether these enzymes are differentially regulated in high and low NUE genotypes. 

## 4. Materials and Methods 

### 4.1. Plant Materials and Growth Conditions 

This study used two soft red winter wheat accessions, VA08MAS-369 and VA07W-415, which were previously characterized as high and low NUE accessions, respectively [22]. Plants were grown under greenhouse conditions as described in Alpuerto et al. [22]. Seeds were grown in cell inserts (72 cells/flat) containing Metro-Mix 360 potting soil under natural light at 22 °C day/13 °C night. At the 3-leaf-stage, seedlings were transferred into a vernalization chamber and incubated at 9 °C under 8 h day (80 mmol/m^2^/s)/16 h night for 9 weeks. Vernalized plants were then transplanted into pots (2.4 L, one plant per pot) containing a mixture of 50% (*v/v*) Metro-Mix 360 and 50% (*v/v*) sand and grown in the greenhouse under natural light at 18 °C day/7 °C night. All plants were supplied with a 1/2 Hoagland’s solution without N (50 mL per plant) twice a week. As an N source, 1 mM of ammonium nitrate was included in the 1/2 Hoagland’s solution. After the jointing stage, two rates of ammonium nitrate (normal N, 1 mM; low N, 0.2 mM) were provided with a 1/2 Hoagland’s solution twice a week until harvest. The light period and temperature were also changed to 16 h day (22 °C)/8 h night (13 °C) to stimulate flowering. 

For root gene expression analysis, plants were grown in a hydroponic system in a growth chamber. Plants were initially grown under 12 h day (400 mmol/m^2^/s; 22 °C)/12 h night (13 °C) for 7 days and then exposed to vernalization as described above. Vernalized plants were incubated under 16 h day (400 mmol/m^2^/s; 22 °C)/8 h night (13 °C) until harvest. A hydroponic solution, including a 1/2 Hoagland’s solution without N and 1 mM of ammonium nitrate, was changed twice a week. After the jointing stage, two rates of ammonium nitrate (normal N, 1 mM; low N, 0.2 mM) were provided with a 1/2 Hoagland’s solution when the hydroponic solution was changed twice a week. Root tissues were collected 0, 5, or 10 days after anthesis, immediately frozen in liquid nitrogen, and stored at −80 °C until use. 

### 4.2. N Measurements

The concentration of N in dried tissues was quantified by combustion analysis following the method of Kim et al. [39]. Grain protein content was calculated by multiplying the grain N content by 6.15, a conversion value for soft red winter wheat.

### 4.3. NUE Index Analysis 

Grain yield was calculated as grain weight (g) per plant. Nitrogen use efficiency (NUE) indexes were computed as follows. NUE for Yield (NUEY) = Grain yield (g)/N supplied (g); NUE for Protein (NUEP) = Grain protein content (g)/N supplied (g); N-uptake efficiency (NUpE) = Above-ground N content (g)/N supplied (g); N-utilization efficiency (NUtE) = Grain yield (g)/Above-ground N content (g) [23,40]. 

### 4.4. Root Morphology Analysis

For root morphology analysis, we used the “cigar roll method” as described in Zhu et al. [24]. Briefly, four-day-old seedlings were placed between two sheets of germination paper and rolled vertically. The rolls were stood up with seedlings upward in a plastic box containing a 1/2 Hoagland’s solution without N plus 1 mM of ammonium nitrate. After three days of incubation, the nutrient solution was replaced with a 1/2 Hoagland’s solution containing 1 or 0.2 mM of ammonium nitrate, and the seedlings were incubated for an additional 6 days. These experiments were performed in a growth chamber under 16 h day (400 mmol/m^2^/s; 22 °C)/8 h (13 °C) night. Root morphology parameters were quantified using WinRHIZO 2017a (Regent Instrument Inc. Canada).

### 4.5. Gene Expression Analysis

RNA extraction and qRT-PCR analysis were performed according to the protocols described by Fukao et al. [41]. Primer sequences used for qRT-PCR are listed in Table S1. 

### 4.6. Statistical Analyses

Statistical analyses were performed using JMP Pro 14 (SAS Institute). Student’s t-test was carried out to compare the mean values between the two genotypes. For multiple mean comparisons, a two-way ANOVA with Tukey’s honest significant difference test was performed. 

## Data Availability

The data presented in this study are available on request from the corresponding authors.

## Supplementary Materials

The following are available online at https://www.mdpi.com/2223-7747/10/1/165/s1, Figure S1. Effect of nitrogen application on yield and yield-related components in two wheat accessions, VA08MAS-369 and VA07W-415, grown in a greenhouse. Table S1. Primer sequences used for qRT-PCR analysis.
