# Supplementary material for: Physiological and Molecular Traits Associated with Nitrogen Uptake under Limited Nitrogen in Soft Red Winter Wheat
Source: Plants (Basel). 2021 Jan 17;10(1):165. doi: 10.3390/plants10010165 (PMC7830070; doi:10.3390/plants10010165)
Supplement: Supplementary file 1 [file plants-10-00165-s001.zip › Figure S1.pdf]

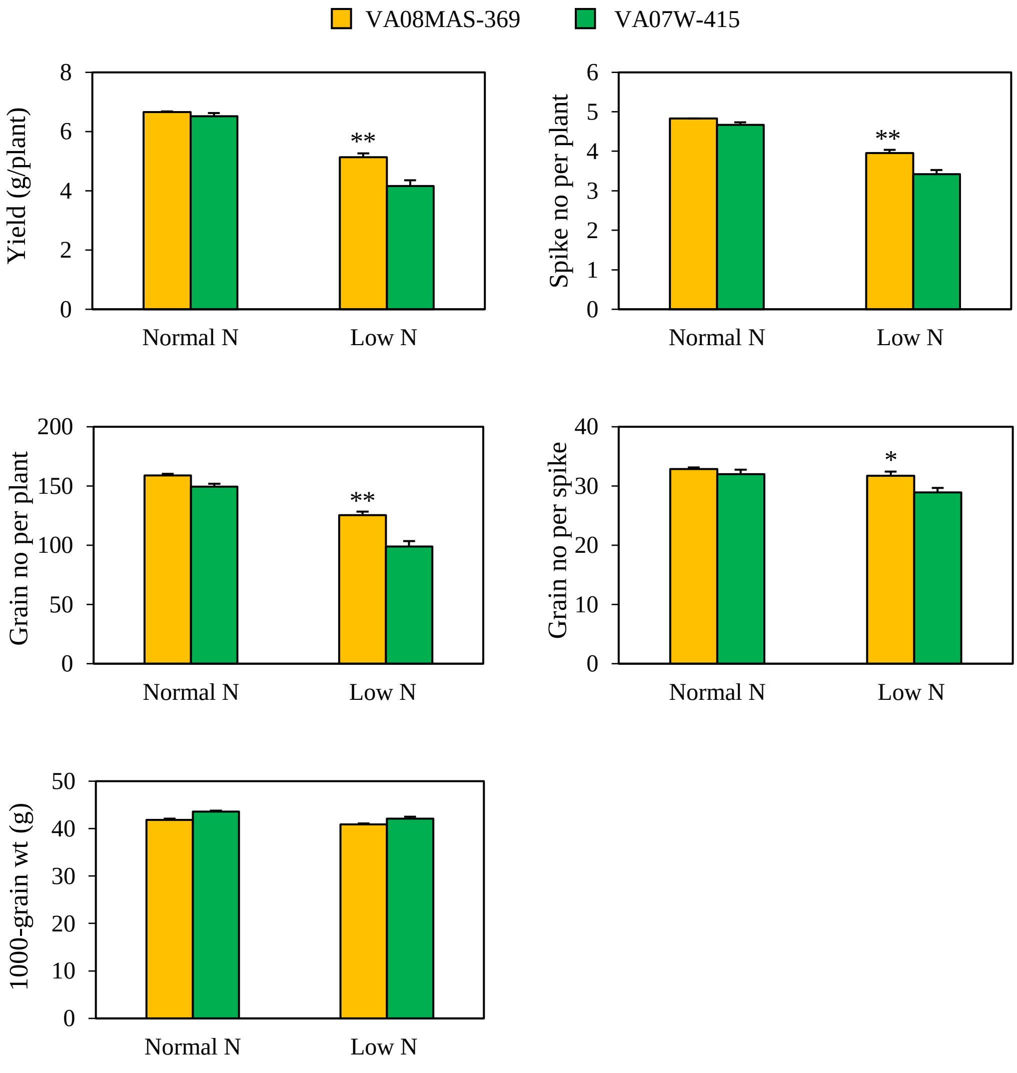

Figure S1. Effect of nitrogen application on yield and yield-related components in two wheat accessions, VA08MAS-369 and VA07W-415, grown in a greenhouse. Data represents means ± SE (n = 4). *P < 0.05, **P < 0.01.
