# Supplementary material for: Physiological and Molecular Traits Associated with Nitrogen Uptake under Limited Nitrogen in Soft Red Winter Wheat
Source: Plants (Basel). 2021 Jan 17;10(1):165. doi: 10.3390/plants10010165 (PMC7830070; doi:10.3390/plants10010165)
Supplement: Supplementary file 1 [file plants-10-00165-s001.zip › Table S1.pdf]

Table S1. Primer sequences used for qRT-PCR analysis.

Primer name	Primer sequence	Reference
NRT2.1-F	GCTGCTTGTGGTGCTGTAT	Wang et al., 2020
NRT2.1-R	GAGCGACGGGTAATGTGC	
NRT2.2-F	CCTCACTAAGCGTGACATCAGC	Wang et al., 2020
NRT2.2-R	CGAGGAGGTCGCACACCAC	
NRT2.3-F	GCTGGGTCTTCGTGCTCCT	Wang et al., 2020
NRT2.3-R	GGCGATGACATTGTCGGTG	
NRT2.4-F	CTGCCCGTCGCCTTCAT	Wang et al., 2020
NRT2.4-R	CGGTCCACTCCCCGTTGTAG	
NRT2.5-F	CGGTGGACTCCGACAACAA	Wang et al., 2020
NRT2.5-R	GAGAACCACGAGAGGTGGAAG	
NRT2.6-F	TTCGGCATCGTTCCCTTC	Wang et al., 2020
NRT2.6-R	TCCTCCACCGTGTACCTGG	
NAR2.1-F	CACGGTCGGAGCTGGTCA	Wang et al., 2020
NAR2.1-R	TGGCTCGCGTTCAGGG	
NAR2.2-F	GGCGGCAGGGTCGAGTA	Wang et al., 2020
NAR2.2-R	GGTGACGCCCGTGATGC	
NAR2.3-F	GGCTCGCCAAGGCATGGTC	Wang et al., 2020
NAR2.3-R	AGGTCGTCGTGGGTCTTGC	
NPF2.1-F	CGGTGGTACAGGTTCAAGAAGTC	Buchner et al., 2014
NPF2.1-R	CGGAGTATAKGAAGCCCCAAAAG	
NPF2.2-F	GTGGACCTRTTCTACCTTGTCAC	Buchner et al., 2014
NPF2.2-R	AGCCTTCTTGGGGCTTTCATCG	
NPF6.1-F	CAATCGGACGGCCTTGATTTCTTC	Buchner et al., 2014
NPF6.1-R	ACGTAGGCACACGTGGACGAC	
NPF6.2-F	ATCGATGCATGCTACTTGCGSTTC	Buchner et al., 2014
NPF6.2-R	GCCACCGAATACACACAAAAACAAG	
NPF7.1-F	CTACAAGACCTGCGCCATCTTC	He et al., 2015
NPF7.1-R	GATGAGGTATAGCCGCGAGGAG	
NPF7.2-F	CCCAGCAGTCAAAGCAAACACTG	He et al., 2015
NPF7.2-R	GGAGGAACACCACCAGGTTCAC	
AMT6-F	GGACTACCTGTGCAACCAGTTC	Bajgain et al., 2018
AMT6-R	CGAAGCCGAAGAGGTAGTAGAA	
AMT49-F	ATGGCTAACACAGAAGCATCAA	Bajgain et al., 2018
AMT49-R	TTTCTTTCGTGGCCTCTACTTC	
AMT52-F	CAGTATGGCAATGGAGAGTTCA	Bajgain et al., 2018
AMT52-R	CACCGATATATGCATTTTGTGC	
EF-α-F	GGTTAAGATGATTCCCACCAAGCC	Wang et al., 2020
EF-α-R	GACAACACCAACAGCAACAGTCTG	
Actin3 like-F	GACGCACAACAGGTATCGTGTTG	Buchner et al., 2014
Actin3 like-R	AGCGAGGTCAAGACGAAGGATG	

References

Bajgain, P.; Russell, B.; Mohammadi, M. Phylogenetic analyses and in-seedling expression of ammonium and nitrate transporters in wheat. *Sci. Rep.* **2018**, *8*, 7082.

Buchner, P.; Hawkesford, M.J. Complex phylogeny and gene expression patterns of members of the NITRATE TRANSPORTER 1/PEPTIDE TRANSPORTER family (NPF) in wheat. *J. Exp. Bot.* **2017**, *65*, 5697-5710.

He, X.; Qu, B.; Li, W.; Zhao, X.; Teng, W.; Ma, W.; Ren, Y.; Li, B.; Li, Z.; Tong, Y. The nitrate-inducible NAC transcription factor TaNAC2-5A controls nitrate response and increases wheat yield. *Plant Physiol.* **2015**, *169*, 1991-2005.

Wang, M.; Zhang, P.; Liu, Q.; Li, G.; Di, D.; Xia, G.; Kronzucker, H.J.; Fang, S.; Chu, J.; Shi, W. TaANR1-TaBG1 and TaWabi5-TaNRT2s/NARs link ABA metabolism and nitrate acquisition in wheat roots. *Plant Physiol.* **2020**, *182*, 1440-1453.
